# Data supporting functional diversity of the marine bacterium *Cobetia amphilecti* KMM 296

**DOI:** 10.1016/j.dib.2016.06.034

**Published:** 2016-06-28

**Authors:** Larissa Balabanova, Olga Nedashkovskaya, Anna Podvolotskaya, Lubov Slepchenko, Vasily Golotin, Alexey Belik, Ludmila Shevchenko, Oksana Son, Valery Rasskazov

**Affiliations:** a G.B. Elyakov Pacific Institute of Bioorganic Chemistry, Far Eastern Branch, Russian Academy of Sciences, Vladivostok, Russian Federation; b School of Economics and Management, Far Eastern Federal University, Vladivostok, Russian Federation

## Abstract

Data is presented in support of functionality of hyper-diverse protein families encoded by the *Cobetia amphilecti* KMM 296 (formerly *Cobetia marina* KMM 296) genome (“The genome of the marine bacterium *Cobetia marina* KMM 296 isolated from the mussel Crenomytilus grayanus (Dunker, 1853)” [1]) providing its nutritional versatility, adaptability and biocontrol that could be the basis of the marine bacterium evolutionary and application potential. Presented data include the information of growth and biofilm-forming properties of the food-associated isolates of *Pseudomonas, Bacillus, Listeria, Salmonella* and *Staphylococcus* under the conditions of their co-culturing with *C. amphilecti* KMM 296 to confirm its high inter-species communication and anti-microbial activity. Also included are the experiments on the crude petroleum consumption by *C. amphilecti* KMM 296 as the sole source of carbon in the presence of sulfate or nitrate to ensure its bioremediation capacity. The multifunctional *C. amphilecti* KMM 296 genome is a promising source for the beneficial psychrophilic enzymes and essential secondary metabolites.

**Specifications Table**

**Table:** 

Subject area	Biology, Chemistry
More specific subject area	Microbiology, Molecular Biology
Type of data	Table, text, figures
How data was acquired	Antimicrobial and hydrocarbon-degrading activities were determined using tests 3 M Petrifilm (USA), crystal violet, plate reader (BioRad), HPLC Shimadzu Prominence (Japan), C-18 column (Supelco).
Data format	Raw, analyzed
Experimental factors	Foodborne bacterial isolates were co-cultured with *C. amphilecti* KMM 296, and their biofilms were treated by *C. amphilecti* KMM 296 metabolites
Experimental features	Testing the biological activity of *C. amphilecti* KMM 296 metabolites on the bacterial cell growth and biofilm-forming properties, and biodegradation of aromatic hydrocarbons
Data source location	Collection of Marine Microorganisms (KMM) at the G.B. Elyakov Pacific Institute of Bioorganic Chemistry FEB RAS,Vladivostok, Russia
Data accessibility	Data is within this article

**Value of the data**

• Data provides new phenotypic characteristics for the description of a new marine bacterial species, *Cobetia amphilecti* [2].
• Data supports the possibility of inducing of previously described *C. amphilecti* KMM 296 genes [1] that are known to be involved in different biochemical pathways in response to the change of environmental conditions such as nutrient medium or microbial competitors, leading to change phenotypic characteristics and to significantly higher survival of the marine bacterium.
• Data includes information on species-dependent decreasing of bacterial cells and biofilms growth under co-culturing conditions with *C. amphilecti* KMM 296 whose metabolites may be of interest for food industry and medicine protection approaches particularly against *P. aeruginosa*.
• Data supports hydrocarbon-degrading activity in *C. amphilecti* KMM 296 in the presence of crude petroleum that may be used in bioremediation of soils and waters.

## 1 Data

**Fig. 1:**
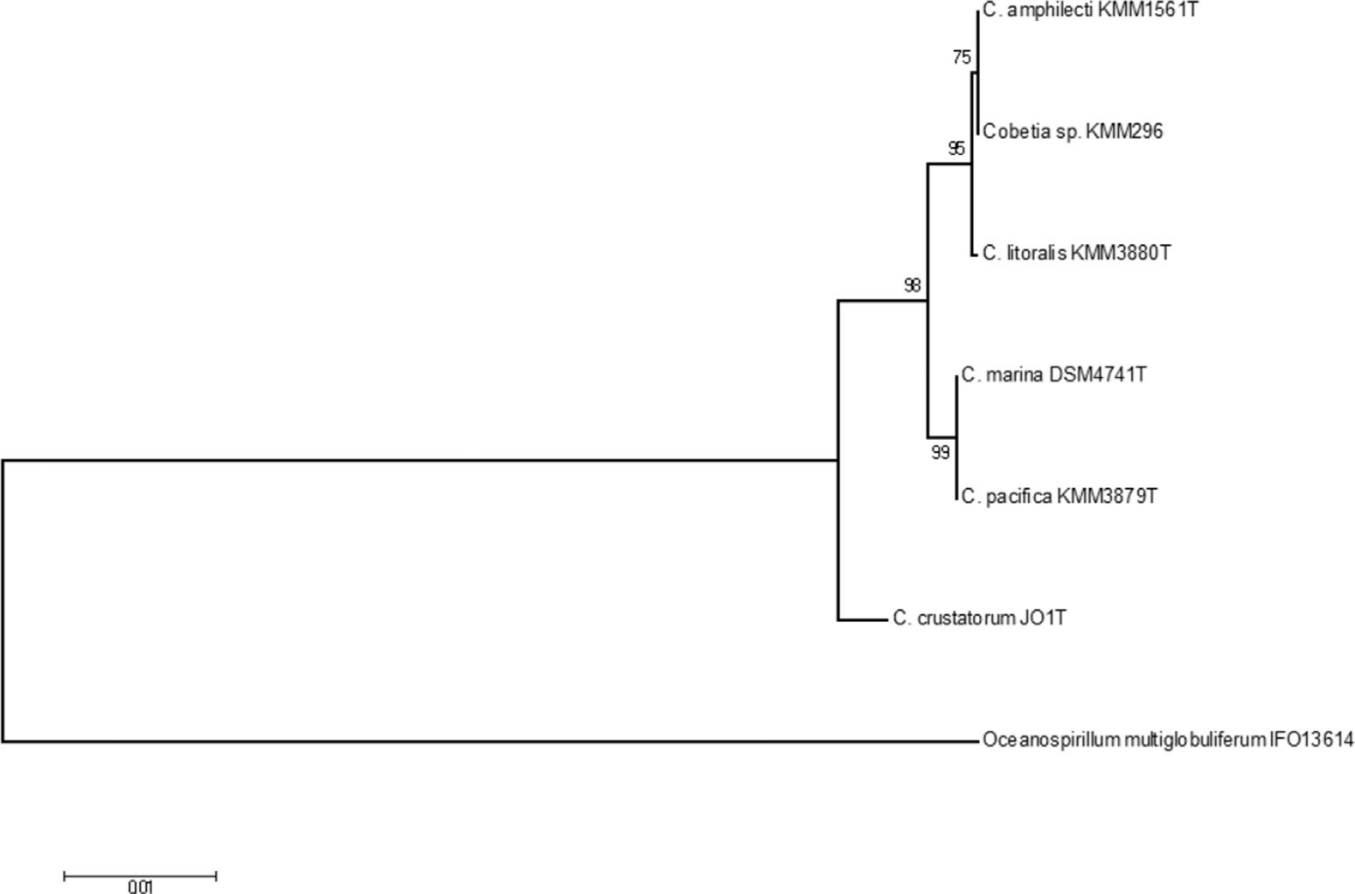
The neighbor-joining phylogenetic tree based on 16 S rRNA gene sequences of *C. amphilecti* KMM1561T (AB646236), *C. litoralis* KMM3880T (AB646234), *C. marina* DSM4741T (AJ306890), C. pacifica KMM3879T (AB646233), *C. crustatorum* (EU909460), showing a position of strain *C. amphilecti* KMM 296 (AY628693) within the genus *Cobetia. Oceanospirillum multiglobiliferum* IFO 13614T (AB006764) was used as outgroup. Bar 0.01 substitutions per nucleotide position.

**Fig. 2:**
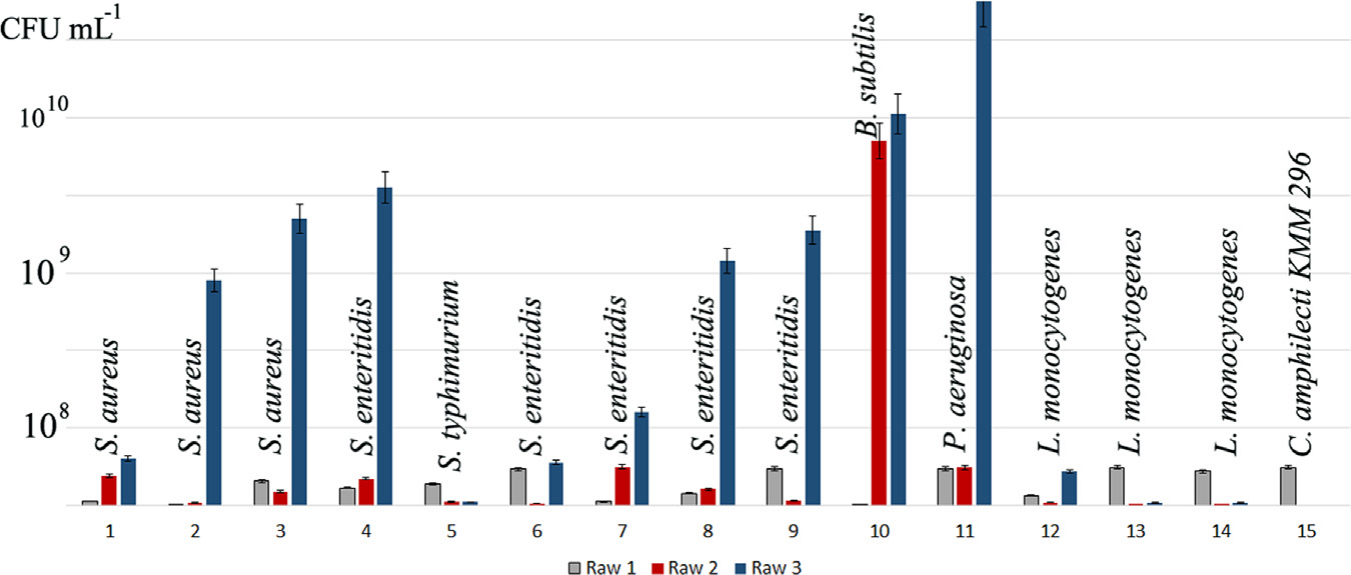
Microbial competition under co-culturing conditions of *C. amphilecti* KMM 296 together with the foodborne bacterial strains for 96 h in the liquid medium containing (g/L): bacto peptone – 2.0 g, casein hydrolyzate – 2.0 g, bacto yeast extract – 2.0 g, dextrin – 1.0 g, KH_2_PO_4_ – 0.02 g, MgSO_4_×7H_2_O – 0.005 g, natural sea water – 500 ml, distilled water – 500 ml, pH – 7.0. Bacterial isolate numbers are on the axis *X*. Cell number quantified in CFU mL^−1^ are on the axis *Y*. Row 1 – cell number of *C. amphilecti* KMM 296 from co-culturing mixes (n.15 – control sample of *C. amphilecti* KMM 296 culturing alone in the same conditions); Row 2 – cell number of the foodborne bacterial isolates from co-culturing mixes; Row 3 – cell number of the foodborne bacterial isolates culturing alone in the same conditions.

**Fig. 3:**
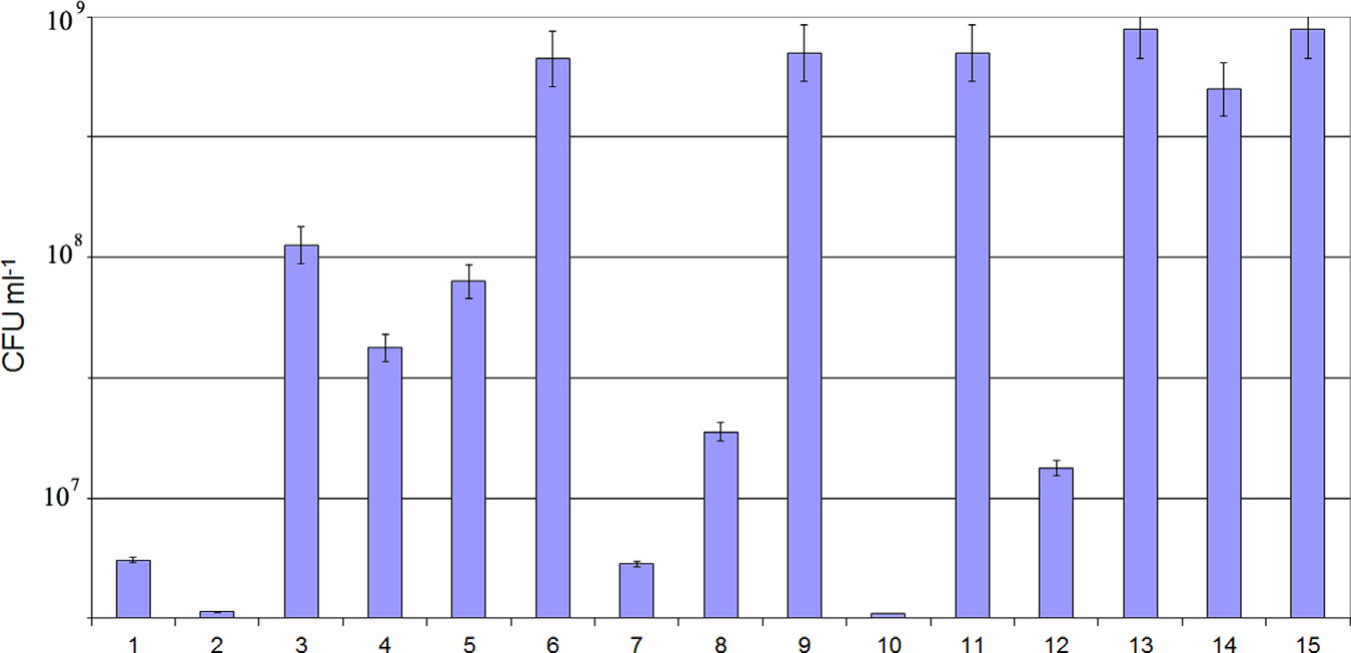
*C. amphilecti* KMM 296 cell density after co-culturing with the foodborne bacterial isolates (15 – control sample of *C.amphilecti* KMM 296 culturing alone in the same conditions). The foodborne bacterial isolate numbers are on the axis *X*. Cell number quantified in CFU mL^−1^ are on the axis *Y*.

**Fig. 4:**
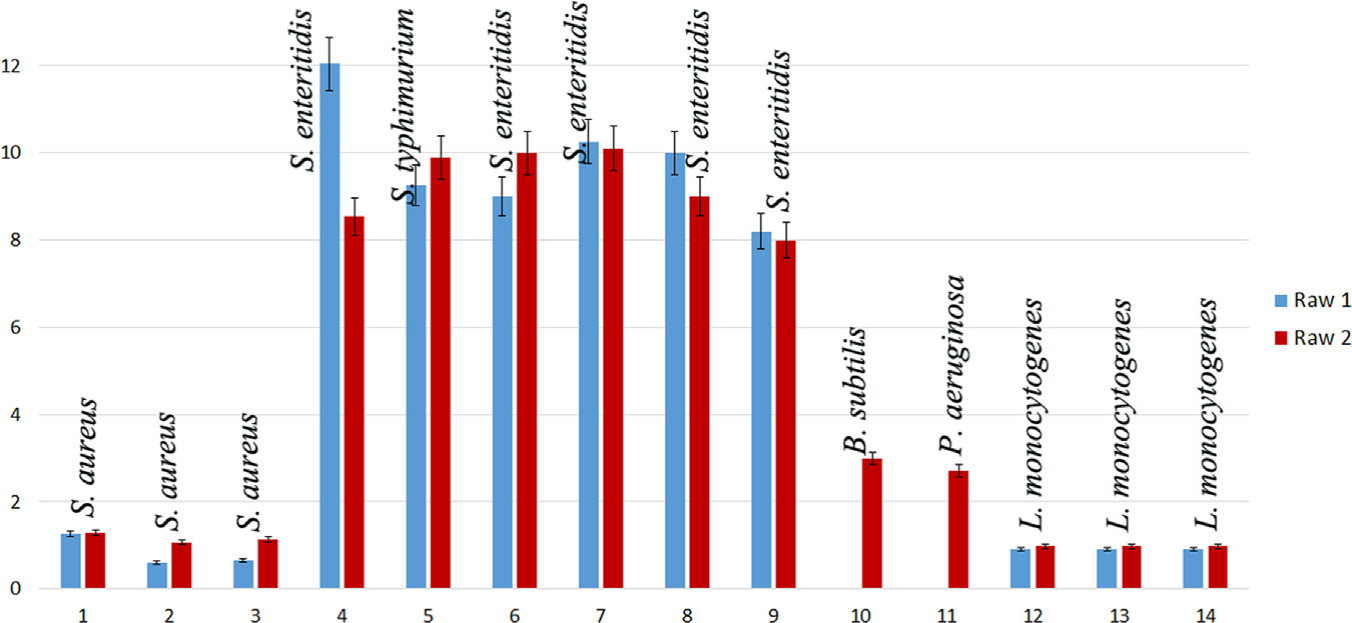
Effect of *C. amphilecti* KMM 296 metabolites on the biofilm density of the food-associated bacterial isolates. Bacterial isolates numbers are on the axis *X*. Coefficient of biofilm density is on the axis *Y*. Row 1 – foodborne bacterial biofilms after the treatment by *C. amphilecti* KMM 296 filtrate from the co-culturing with the same species. Row 2 – control samples in the same conditions of incubation.

The mussel-associated isolate of the marine bacterium KMM 296 (formerly *C. marina* KMM 296) [1], [2], [3], [4] was identified as the species *C. amphilecti* according to the 16 S rRNA-based analysis (Fig. 1) with 100% sequence identity with the type strain of recently described species of the genus *Cobetia* [2]. Although KMM 296 did not exhibit some enzymatic activity in the standard conditions (Table 1), it was able to consume petroleum hydrocarbons as the sole source of carbon in the presence of sulfate or nitrate (4 g/L) after 3–6 days of cultivation at aerobic conditions. The filtrate of *C. amphilecti* KMM 296 cultivated alone had no any noticeable effect on the bacterial isolates, but their presence in the medium for co-culturing resulted in its inhibition effect, lowering their colony-forming units (CFU mL^−1^) from 2 to 1000-fold, and degrading completely the *B. subtilis* and *P. aeruginosa* biofilms during 96 h of incubation (Fig. 2, Fig. 3, Fig. 4).

## 2 Experimental design, materials and methods

### 2.1 Morphological, biochemical, and physiological characterization

The physiological, morphological and biochemical properties of the marine bacterial strain KMM 296 were studied using the standard methods, and the API 20E, API 20NE, API 50 CH, API 32 ID GN and API ZYM galleries (bioMérieux, France). Gram-staining was performed as recommended by Gerhardt et al. [5]. Oxidative or fermentative utilization of glucose was determined on Hugh and Leifson׳s medium modified for marine bacteria [6]. Catalase activity was tested by addition of 3% (v/v) H_2_O_2_ solution to a bacterial colony and observation for the appearance of gas. Oxidase activity was determined by using tetramethyl-*p*-phenylenediamine. Degradation of agar, starch, casein, gelatin, chitin, DNA and urea and production of acid from carbohydrates, hydrolysis of Tween 80, nitrate reduction, production of hydrogen sulfide, acetoin (Voges-Proskauer reaction) and indole were tested according to the standard methods [5]. The temperature range for growth was assessed on Marine agar. Tolerance to NaCl was assessed in medium containing 5 g bacto peptone (Difco), 2 g bacto yeast extract (Difco), 1 g glucose, 0.02 g KH_2_PO_4_ and 0.05 g MgSO_4_×7H_2_O per liter of distilled water with 0, 0.5, 1.0, 1.5, 2.0, 2.5, 3, 4, 5, 6, 8, 10, 12, 15, 17, 19, and 20% (w/v) of NaCl. Susceptibility to antibiotics was examined by the routine disc diffusion plate method. Discs were impregnated with the following antibiotics: ampicillin (10 μg), benzylpenicillin (10U), carbenicillin (100 μg), cefalexin (30 μg), cefazolin (30 μg), chloramphenicol (30 μg), erythromycin (15 μg), gentamicin (10 μg), kanamycin (30 μg), lincomycin (15 μg), nalidixic acid (30 μg), neomycin (30 μg), ofloxacin (5 μg), oleandomycin (15 μg), oxacillin (10 μg), polymyxin B (300 U), rifampicin (5 μg), streptomycin (30 μg), tetracycline (5 μg) and vancomycin (30 μg).

### 2.2 Co-culturing assay

The strain KMM 296 and fourteen isolates of *Pseudomonas, Bacillus, Listeria, Salmonella* and *Staphylococcus* from ready-to-cook meat foods were used. All bacterial strains were isolated and identified to the species level by standard microbiological methods in the accredited laboratory (ISO/IEC 17025). The food-associated isolates were cultivated on the appropriate strain-specific nutrient mediums. Strain KMM 296 was cultivated for 12 h on a nutrient medium containing (g/L): bacto peptone – 2.0 g, hydrolyzed casein – 2.0 g, bacto yeast extract – 2.0 g, dextrin – 1.0 g, KH_2_PO_4_ – 0.02 g, MgSO_4_×7H_2_O – 0.005 g, agar – 15 g, natural sea water – 500 mL, distilled water – 500 mL, pH – 7.0.

The colonies of the pure culture of every bacterial isolate were suspended in 0.9% NaCl until they matched a McFarland turbidity of 0.5 (1×10^8^ CFUmL^−1^). After that, 2 mL of the bacterial isolate and 2 mL of *C. amphilecti* KMM 296 suspensions were simultaneously inoculated in 16 mL of the nutrient medium described above. The strains cultivated separately in the same conditions were served as controls. Incubation was performed on shaker at 200 rpm for 48, 72 and 96 h at the temperature 28 °C, and then the cell numbers were calculated. To check the cell number, a 200 µL of the inoculum was serially tenfold diluted from 10^−1^ to 10^−8^ CFUmL^−1^ and applied for the strain-specific grid test 3 M Petrifilm (USA). Four 10 μL drops from each dilution of the *C. amphilecti* KMM 296 cell suspension was spotted on the Marine agar. Colony formation was assessed after 24 h.

### 2.3 Antibiofilm activity assay

Antibiofilm activity was determined by semi-quantitative adherence assay in sterile U-bottom 96-well polystyrene microtiter plates (Greiner Bio-One) [7]. 100 µL suspensions of the overnight bacterial culture of the foodborne isolate grown in tryptic soy broth (TSB, Merc) with the density 1×10^8^ CFUmL^−1^ were loaded into the wells, and then 100 µL of the filtrate of three-day grown *C. amphilecti* KMM 296 alone or after co-culturing with the same bacterial isolate was added. In each of the wells, 100 µL 1% peptone was added. The sterile medium for *C. amphilecti* KMM 296 cultivation and the bacterial cells with 1% peptone were served as controls. The plates were incubated for 2-5 days at the room temperature. The plankton cells were removed and cell number was determined as described above. The biofilm was further assessed by using the crystal violet (CV) assay. The procedure involved washing the plates three times with sterile 0.85% NaCl solution to remove loosely associated cells. Then, 200 µL of 0.5% CV water solution was added in each well and incubated for 15 min at the room temperature, after that the plates were washed 3-4 times with sterile distilled water to remove unabsorbed stain, and then the plates were air-dried. For the quantities determination of biofilm biomass, 200 µL of 96% ethanol containing 2% acetic acid was added in each well, incubated at the room temperature for 15 min, and the optical density at 600 nm was measured with an automatic plate reader (BioRad). The biofilm adherence or detachment of each isolate was measured in triplicate considering the coefficient of biofilm accretion (OD_sample_/OD_control_).

### 2.4 Hydrocarbon-degrading activity assay

The degradation capabilities of *C. amphilecti* KMM 296 towards hydrocarbons were carried out by growing the cells (10^6^–10^7^ CFUmL^−1^) in 5 mL of depleted medium containing 0.05 mL sterile crude petroleum as a sole source of carbon, K_2_HPO_4_ – 1.0 g/L, KH_2_PO_4_ – 1.0 g/L, MgSO_4_×7 H_2_O – 0.2 g/L, CaCl_2_ – 0.02 g/L, NaCl – 20.0 g/L, FeCl_3_ saturated solution – 0.05 mL/L, and nitrate or sulfate ammonium in various concentrations: (1) NH_4_NO_3_ – 1.0 g/L; (2) (NH_4_)_2_SO_4_ – 1.0 g/L; (3) (NH_4_)_2_SO_4_ – 2.0 g/L; (4) (NH_4_)_2_SO_4_ – 4.0 g/L, and distilled water, pH 7.0-7.3. The same sterile mediums were used as controls. Incubation was performed on rotor shaker at 200 rpm/min, 22–24 °C for 3, 6, 10 and 16 days. The HPLC analysis of the crude petroleum products was carried out by using column C-18 (Supelco). Hydrophobic fractions were separated and dissolved in methanol or acetonitrile, or their water mixtures (v/v) (1:1; 3:1). The extracts were filtered and analyzed with the use of the same dissolvent as mobile phase at a flow rate of 0.5 mL min^−1^. UV-detector was set at 254 nm for compounds detection. The HPLC solvent delivery system was Shimadzu Prominence (Japan). Injection volume was 100 µL.

### 2.5 Phylogenetic analysis

The neighbor-joining (NJ) phylogenetic tree was constructed by MEGA v.6 [8]. 16 S rRNA gene sequences of *C. amphilecti* KMM1561^T^ (AB646236), *C. litoralis* KMM3880^T^ (AB646234), *C. marina* DSM4741^T^ (AJ306890), *C. pacifica* KMM3879T (AB646233), *C. crustatorum* (EU909460), *C. amphilecti* (formerly *C. marina*) KMM 296 (AY628693), and *Oceanospirillum multiglobiliferum* IFO 13614 T (AB006764) as the outgroup were used.

### 2.6 Statistical analysis

All values presented in this article are representative of at least three independent experiments. Data were analyzed using the Student׳s t-test of the SigmaPlot 2000 version 6.0 program (SPSS Inc.). Differences from controls were considered significant at P ≤ 0.05.

## Conflict of interest

None.

## Figures and Tables

**Table 1:** Differential characteristics of strain KMM 296 and *Cobetia amphilecti* KMM 1561^T^.

**Characteristic**	**KMM 296**	**KMM 1561** **^T^**⁎
Source and site of isolation	Mollusc *Crenomytilus grayanus,* the Sea of Japan, Pacific Ocean	Sponge *Amphilectus digitatus*, the Gulf of Alaska, Pacific Ocean
Oxidase	+	−
Salinity range (% NaCl)	0.5-19	0-20
Hydrolysis of DNA	−	+
		
Acid production from:		
>d-Cellobiose, d-fructose, d-galactose, d-glucose, d-lactose, maltose, d-xylose, inositol	−	+
		
Assimilation of:		
Maltose, d-gluconate, l-malate, l-rhamnose, N-acetylglucosamine, d-ribose, inositol, suberic acid, lactic acid, l-alanine, potassium 5-ketogluconate, 3-hydroxybenzoic acid, l-serine, salicin, melibiose, l-fucose, l-arabinose, l-histidine, potassium 2-ketogluconate	–	+
Capric acid, valeric acid	+	−
		
Enzyme activities:		
Naphthol-AS-BI-phosphohydrolase, valine arylamidase, β-galactosidase	−	+
Trypsin	+	−
		
Susceptibility to:		
Rifampicin, tetracycline and erythromycin	+	−
DNA G+C content (mol%)	62.7⁎⁎	63.4

**Footnote:** Both strains were positive for the following tests: respiratory type of metabolism, motility; slightly yellowish colony color; growth at 4–42 °C; catalase, alkaline phosphatase, esterase (C4), esterase lipase (C8), leucine arylamidase, acid phosphatase and α-glucosidase activities, PNPG test; assimilation of sucrose, maltose, sodium malonate, glycogen, d-mannitol, d-glucose, 3-hydroxybutyric acid and l-proline, susceptibility to carbenicillin, cephalexin, cephazolin, chloramphenicol, gentamicin, kanamycin, nalidixic acid, neomycin, ofloxacin, polymyxin B, streptomycin and vancomycin; and resistance to ampicillin, benzylpenicillin, lincomycin, oleandomycin and oxacillin. Both strains were negative for the following tests: arginine dihydrolase, lipase (C14), cystine arylamidase, α-chymotrypsin, N-acetyl-β-glucosaminidase, β-glucosidase, α-galactosidase, β-glucuronidase, α-mannosidase and α-fucosidase activities, hydrolysis of agar, chitin, aesculin, gelatin, starch, urea and Tween 80; acid production from d-mannose, melibiose, raffinose, l-rhamnose, d-ribose, N-acetylglucosamine, inositol, d-sorbitol, glycerol and d-mannitol; nitrate reduction; production of H2S and indole; assimilation of l-arabinose, d-mannose, N-acetylglucosamine, adipate, phenylacetate, itaconic acid, sodium acetate, propionic acid, trisodium citrate and 4-hydroxybenzoic acid.

⁎ , Data from Romanenko et al. [2].

⁎⁎ , Data from Ivanova et al. [3].
