# Supplementary material for: Data supporting functional diversity of the marine bacterium Cobetia amphilecti KMM 296
Source: Data Brief. 2016 Jun 28;8:726–32. doi: 10.1016/j.dib.2016.06.034 (PMC4949733; doi:10.1016/j.dib.2016.06.034)
Supplement: Supplementary file 1 — Supplementary material [file mmc1.docx]

There are no conflicts of interests.
